# Bacterial Diarrhcea at Leicester

**Published:** 1889-08-10

**Authors:** 


### Within the Wards.

### BACTERIAL DIARRHŒA AT LEICESTER.

The town of Leicester has for a good many years enjoyed an unenviable notoriety for the high mortality of its inhabitants from diarrhœa. The disease, besides being prevalent in certain limited districts of the town, has also coincided with other well-defined conditions—*e. g.*, high summer temperature, poverty and feebleness of constitution in those attacked, and so on. It is obvious that when for a long period, of nearly forty years, a disease has been prevalent in one place, and always under like conditions, there must be certain more or less uniform causes at work in the production of it, and those causes, being thus localised and indicated by the regularity of the recurring circumstances, ought to be comparatively easy of discovery. Acting on the indications manifested, the medical officer of health for the borough, Dr. Henry Tomkins, B.Sc., has made careful and interesting experiments with the view of determining the part played in the production of the diarrhœa by the air and soil respectively. He was led to examine the air and the soil more especially because the water and food seemed to be excluded as causes by the facts that the water supply of the affected districts was also the water supply of the whole town, and the food commonly used in these districts was similar to that commonly used by the same classes in districts not affected. The object of the experiments was to determine the number of disease-producing bacteria in a measured quantity of the air of the affected districts as contrasted with the number found in the same quantity of air in the free districts. Similar experiments with the same object were made with the soil of the affected and the non-affected areas.

The part of the town where the disease almost exclusively prevailed is the low-lying one following the River Soar, on which Old Leicester was built. The older and poorer houses and streets are situated in that area; the new town and the wealthier inhabitants having gradually taken possession of the higher and remoter undulations of the ground. Within what Dr. Tomkins calls the "diarrhœa area," it is important to note that the disease attacks all classes of people indiscriminately, sparing neither age nor sex, prosperous nor poor. It is, however, much the most fatal among children, the maximum fatality being reached in infants under one year old. All the circumstances go to show that for fifty years past the low-lying and poorest parts of Leicester have been the abode of a more or less continuous and fatal form of diarrhœa, exaggerated by hot weather, checked somewhat by cold, but never wholly absent or harmless. What, asks Dr. Tomkins, is the definite and unquestioned cause of this state of things? Two causes, he thinks, he has certainly discovered—viz., the air and the soil. In examining the air he set himself to demonstrate by comparison experiments the number of microbes found in a measured quantity taken from the infected area, as contrasted with the number found in the same quantity taken from non-infected areas. He employed the cultivation method with nutrient gelatine, which has been before described in these pages, and with which bacteriologists are perfectly familiar. The results obtained showed that within the affected area itself the atmosphere contains double or treble the number of bacteria during the prevalence of the disease that it contains at ordinary times. Hot weather almost invariably causes an exacerbation both in the number and severity of the cases, whilst cold weather as constantly produces an abatement of the severer indications. The contrast between the number of microbes found in the same quantity of air within and without the affected area was still more marked. Within the area, each cubic metre of air contained from 6,000 to 7,000 microbes; outside, the same quantity contained only from 600 to 1,400. Not only so, but the cultivations obtained from the air of the infected area were much more rapid and complete, and the odour from them was much more offensive. Experiments made with soils taken first from the "diarrhœa area," and then from districts outside, demonstrated the important fact that even the ground can be charged with deadly poison to an alarming extent. The soil of the affected parts of the town, varying from six inches to six feet in depth, contained from two to six times as many microbes as the same quantity of soil taken from the ground beyond the radius of infection. Especially was the soil charged with microbes in those parts where, from leakage and other causes, sewage had been permitted to soak the ground.

These are facts of the highest importance. They go to show that the health of towns is largely in the hands of the inhabitants themselves. They confirm all previous experience of the subject; and they point out, both to municipal authorities and private individuals, the clear way of duty and safety. It is incumbent upon municipalities to steadily restrict such disease areas as Dr. Tomkins points out, and to make it a definite object to put an end to their existence. All such portions of towns and cities should be cleared of inhabitants as quickly as possible, and disinfected by being restored to cultivation. That is the only rational and final way of dealing with them. New districts should be effectually prevented from degenerating into such poison areas by enforced public and private sanitation. The poor must be instructed in sanitary laws, and must be made to observe them under penalties. There is no doubt whatever that if responsible authorities would carry out faithfully the teachings of plain and demonstrable science, even our large towns might become almost as free from zymotic diseases as the desert of Sahara or the open ocean.

